# Association of statins, gliptins, and antipsychotics with bullous pemphigoid: A case–control study in the Cretan population

**DOI:** 10.1111/1346-8138.17603

**Published:** 2025-01-08

**Authors:** Eirini Kavvalou, Konstantinos Krasagakis, Gregory Chlouverakis, Paraskevi Xekouki, Vasiliki Daraki, Charikleia Kouvidou, Eleni Lagoudaki, Sabine‐Elke Krüger‐Krasagakis

**Affiliations:** ^1^ School of Medicine University of Crete Heraklion Greece; ^2^ Department of Dermatology University General Hospital of Heraklion Heraklion Greece; ^3^ Department of Endocrinology University General Hospital of Heraklion Heraklion Greece; ^4^ Department of Pathology Evangelismos General Hospital Athens Greece; ^5^ Department of Pathology‐Histology University General Hospital of Heraklion Heraklion Greece

**Keywords:** antipsychotics, bullous pemphigoid, case–control study, gliptins, statins

## Abstract

Bullous pemphigoid (BP) is an autoimmune blistering disorder predominantly affecting the elderly. Recently, many studies have shed light on the effect of specific drug intake and comorbidities on the development of BP. The purpose of this study was to investigate the association of specific drug class intake and comorbidities with the development of BP in the Cretan population. Significant associations with BP were found for statins (odds ratio [OR] = 4.06, 95% confidence interval [CI] 1.99–8.27, *P* < 0.001), gliptins (OR = 4.27, 95% CI 2.33–7.83, *P* < 0.001), and antipsychotics (OR = 3.33, 95% CI 1.36–8.11, *P* = 0.006). Higher proportions of use in the BP group vs. control group were found for atorvastatin (OR = 1.86, 95% CI 1.04–3.32, *P* = 0.035), linagliptin (OR = 6.63, 95% CI 2.17–20.23, *P* < 0.001), vildagliptin (OR = 3.20, 95% CI 1.73–5.91, *P* < 0.001), alogliptin (OR = 5.11, 95% CI 1.19–22.04, *P* = 0.016), and quetiapine (OR = 4.21, 95% CI 1.5–11.85, *P* = 0.004). The presence of diabetes mellitus in the absence of gliptins did not show any significant effect on BP (OR = 1.60, 95% CI 0.79–3.23, *P* = 0.188). Metformin intake showed no significant association with BP (OR = 0.48, 95% CI 0.18–1.28, *P* = 0.143). Our findings confirm and extend previous studies reporting the association of gliptins and antipsychotics on BP in other European populations. The association found for statins is new, thus more studies are needed to corroborate its validity.

## 1 INTRODUCTION

Bullous pemphigoid (BP) is an autoimmune blistering disorder that predominantly affects the elderly. The pathophysiology of BP involves the creation of autoantibodies to self‐antigens in the basement membrane, specifically BP230 and BP180. In a subset of patients with BP, drugs have been implicated in its pathogenesis.^1^ Patsatsi et al.^2^ showed that patients who were receiving systemic medications seemed to be more susceptible to the development of BP. In drug‐induced BP, certain medications presumably act as haptens and induce an antibody response. Haptens can bind to proteins in the lamina lucida and change their antigenic properties, thus stimulating the antibody response.^1^ Recently, several studies have described an association between dipeptidyl peptidase‐4 inhibitors (DPP‐4i), also known as gliptins, and BP development. Kridin et al.^3^ found that an overall exposure to DPP‐4i is associated with an 80% increase in the odds of subsequent BP (odds ratio [OR] = 1.81, 95% confidence interval [CI] 1.46–2.08, *P* < 0.001). Additionally, Douros et al.^4^ showed in a cohort study that the current use of DPP‐4i is associated with an increased risk of BP (47.3 vs. 20.0 per 100 000 person‐years, hazard ratio [HR] 2.21, 95% CI 1.45–3.38).

Only a few studies have reported an association between BP and other drug classes besides gliptins, such as the statins and the antipsychotics. Regarding the association between statins and BP development, this seems to be rather controversial; Papadopoulou et al.^5^ and Rozenblat et al.^6^ showed a protective role of statins, whereas Chang et al.^7^ found no association between statins and BP development. Furthermore, only a few studies have so far investigated the association between antipsychotics and the development of BP, and their results refer to a specific limited population, for example the Finnish population.^8^

Crete is the third largest island in the Mediterranean, with a relatively stable, genetically homogeneous population. There is no study so far examining the relation between different drug classes and BP development in the Cretan population. The aim of our study was to investigate the possible risk of different drug classes, including the previously mentioned, in the development of drug‐induced BP in Cretans.

## 2 METHODS

We conducted a retrospective 1:3 case–control study at the University General Hospital of Heraklion on the island of Crete. The study included 64 cases with BP diagnosis and 191 sex‐ and age (±2 years)‐matched controls. Inclusion criteria for both cases and controls were Cretan descent (up to two generations) and admission to hospital during the period 2012–2020. The diagnosis of BP in cases was determined by clinical features, histopathological diagnosis, and at least one of the following immunological examinations: positive direct immunofluorescence or positive enzyme‐ linked immunosorbent assay (ELISA) serum test.^9^ Controls must have visited the hospital the same year the case was admitted to the hospital because of a BP diagnosis. Exclusion criteria for all participants were the presence of another autoimmune bullous disease and age under 40 years.

Institutional Ethics Committee approval from the University of Crete and from the University General Hospital of Heraklion was sought and obtained (Protocol Numbers 156/30.07.2020 and 1005/33/02‐12‐2020 respectively) and written informed consent from all participants was also obtained.

Collected demographic and clinical data included sex, age, body mass index (BMI), living area, smoking, patient's medical history (arterial hypertension, coronary artery disease [CAD], diabetes mellitus [DM], dyslipidemia, chronic obstructive pulmonary disease, malignancy, atopy, functional thyroid disorder, neurodegenerative disease [dementia, Alzheimer's disease, Parkinson's disease], psychiatric disease, hyperuricemia, autoimmune disease [rheumatoid arthritis, autoimmune thyroiditis, systemic scleroderma, systemic lupus erythematosus, Sjogren's disease, vitiligo, polymyalgia rheumatica and giant cell arteritis], psoriasis, ophthalmologic disease), and drugs taken from 2012 until the onset of symptoms, classified in drug categories. The drug classes examined included the following: proton‐pump inhibitors, β‐blockers, statins, angiotensin receptor blockers, cholinesterase inhibitors, bisphosphonates, angiotensin‐converting enzyme inhibitors, 5‐α‐reductase inhibitors, α‐blockers, calcium channel blockers, antipsychotics, antidepressants, dopaminergic agents, diuretics, H_2_ antagonists, antithrombotic agents, and antidiabetics. To collect these data, we used a standardized questionnaire.

Participants in the control group were randomly selected from patients admitted to the internal medicine department and patients visited the dermatological and endocrinological outpatient clinics.

Discrete and qualitative data are presented as counts and percentages, while continuous data are summarized with mean  and standard deviation. Univariate and multivariate associations between BP and possible risk and preventive factors were expressed in the form of OR with 95% confidence interval (CI). Univariate associations between BP and patients' demographics, chronic diseases, and drug categories were also assesed with ORs. Multivariate stepwise logistic regression models with BP as the dependent variable were utilized to access associations of chronic diseases from patients' medical history that related to BP from univariate analyses and to examine the association of drug categories related to BP from univariate analyses.

Adjusted ORs with 95% CI were computed to determine the magnitude of the effect size of potential risk/protective factors for graphical representation of data. Scatterplots using ORs with 95% CI as measures of dispersion were used for graphical representation of data. IBM SPSS Statistics 24.0 was used for statistical analysis and an *a* = 0.05 was set as level of acceptance.

## 3 RESULTS

A total of 255 people participated in this study in a ratio of 1:3 (patients:controls). There were 64 BP patients and the corresponding control sample consisted of 191 people (Table 1). There were 39 females in the BP group (60.9%) and there was no difference in sex distribution between the control and BP groups (*P* = 0.964). Living area and smoking were adjusted between the two groups, with *P* = 0.400 and *P* = 0.585 respectively. Between the BP and control groups there was no significant difference in age (*P* = 0.861) and BMI (*P* = 0.799).

**TABLE 1:** Baseline demographic characteristics of BP patients and the control group.

		Group		Total	*P*
		Control	BP		
		*n* (%) (191)	*n* (%) (64)		
Sex	Female (156)	117 (61.3)	39 (60.9)	255	0.964
Locations	Rural (159)	122 (65.6)	37 (59.7)	248	0.400
Smoking	No (150)	110 (61.8)	40 (64.5)	240	0.585
	Yes (33)	23 (12.9)	10 (16.1)		0.672
	Ex‐smoker (57)	45 (25.3)	12 (19.4)		0.407
Age (years) mean ± SD		77.1 ± 9.5	77.4 ± 9.6	251	0.861
BMI (kg/m^2^) mean ± SD		28.1 ± 5.7	28.4 ± 6.5	239	0.799

*Note*: *P* is the from Students' *t*‐test or the Pearson chi‐square test.

Abbreviations: BMI, body mass index; BP, bullous pemphigoid; *n* (%) refers to frequency (% frequency); SD, standard deviation.

**FIGURE 1:**
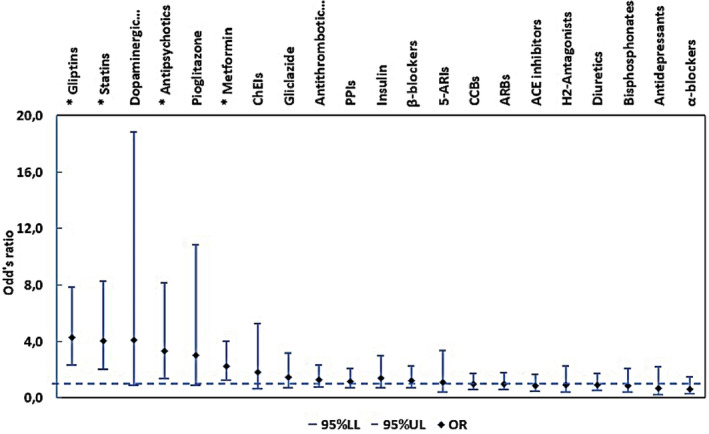
Estimated odds ratios (ORs) with 95% confidence intervals for bullous pemphigoid for each of the studied drug categories presented in the form of scatter with error bars. 95% LL, 95% lower confidence limit of OR; 95% UL, 95% upper confidence limit of OR. The horizontal dash line is OR = 1.0. 5‐ARIs, 5‐α‐reductase inhibitors; ACE, angiotensin‐converting enzyme; ARBs, angiotensin receptor blockers, CCBs, calcium channel blockers; ChEIs, cholinesterase inhibitors; PPIs, proton‐pump inhibitors.

In Figure 1 the effect on BP for each drug category is shown. Estimated ORs with 95% CI resulted from logistic regression analyses. A significant effect on BP development was found for gliptins (OR = 4.27, 95% CI 2.33–7.83, *P* < 0.001), statins (OR = 4.06, 95% CI 1.99–8.27, *P* < 0.001), and antipsychotics (OR = 3.33, 95% CI 1.36–8.11, *P* = 0.006). In these drug categories the proportion of BP patients was higher than in the controls. More specifically, 67.2% of BP patients vs. 31.0% of controls were on gliptins, 82.5% of BP patients vs. 53.8% of controls were on statins, and 17.5% of BP patients vs. 6.0% of controls were on antipsychotics. A detailed description of estimated ORs for BP vs. drug categories can be found in Supporting Information Table S1.

**FIGURE 2:**
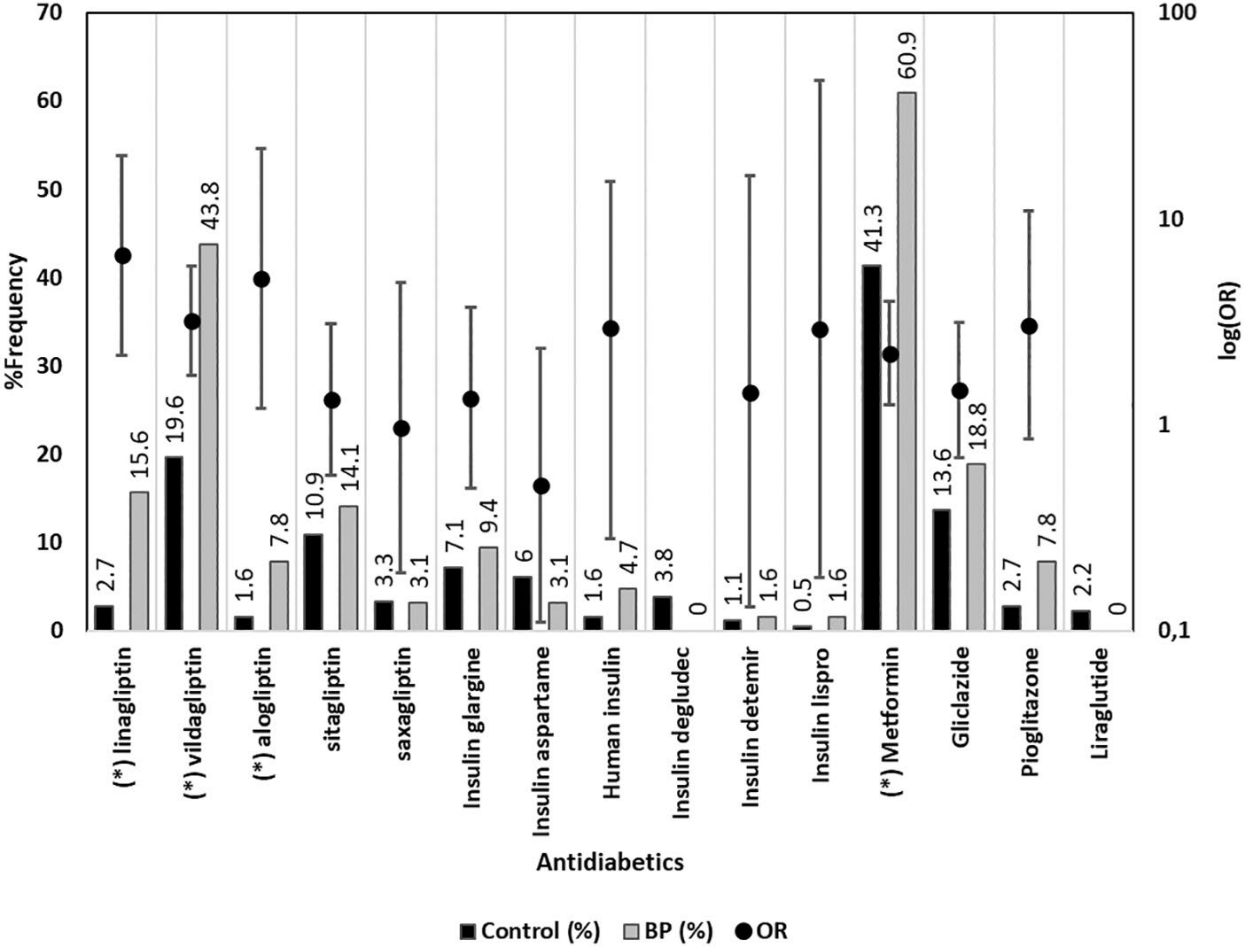
The percentage use of antidiabetics in the control and bullous pemphigoid (BP) groups. Dots with vertical lines are ORs (odds ratio) with 95% confidence intervals in log scale. Asterisks (*) indicate significant differences in antidiabetics use between the two groups.

When each of the drugs was examined for association with BP, higher proportions for atorvastatin use were found in the BP group (31 patients, 49.2%) vs. the control group (63 patients, 34.2%) (OR = 1.86, 95% CI 1.04–3.32, *P* = 0.035). The proportion of quetiapine use was also higher in the BP group (9 patients, 14.3%) vs. the control group (7 patients, 3.8%) (OR = 4.21, 95% CI 1.5–11.85, *P* = 0.004). Linagliptin, vildagliptin, and alogliptin intake was higher in the BP group (linagliptin: 10 patients, 15.6%; vildagliptin: 28 patients, 43.8%; alogliptin: 5 patients, 7.8%) vs. the control group (linagliptin: 5 patients, 2.7%; vildagliptin: 39 patients, 19.6%; alogliptin: 3 patients, 1.6%), and the corresponding ORs were linagliptin 6.63 (95% CI 2.17–20.23, *P* < 0.001), vildagliptin 3.20 (95% CI 1.73–5.91, *P* < 0.001), and alogliptin 5.11 (95% CI 1.19–22.04, *P* = 0.016). A significant difference was found in the proportion of metformin intake in BP patients (39 patients, 60.9%) vs. controls (76 patients, 41.3%), with an estimated OR = 2.22 (95% CI 1.24–3.97, *P* = 0.007). Due to the extensive number of medications included in the analysis, the results can be found in Supporting Information Table S3. A detailed description of antidiabetics use between BP and control groups is shown in Figure 2.

In addition to the previous analysis, a multivariate logistic regression model was applied to establish possible effects of the above‐mentioned drug categories that showed an association with BP in simple logistic regression (gliptins, statins, antipsychotics, and the antidiabetic drug metformin). When drug categories or metformin were included as explanatory variables in the initial model, significant effects were found for statins (OR = 3.06, 95% CI 1.43–6.55, *P* = 0.004), antipsychotics (OR = 4.16, 95% CI 1.52–11.38, *P* = 0.005), and gliptins (OR = 3.32, 95% CI 1.73–6.36, *P* < 0.001), but not for metformin intake (OR = 0.48, 95% CI 0.18–1.28, *P* = 0.143) (Table 2).

**TABLE 2:** Multiple logistic regression model of BP with drug categories and the antidiabetic drug metformin showing an association with BP in the univariate analysis.

	Method = enter	
	OR (95% CI)	*P*
Statins	3.06 (1.43–6.55)	0.004*
Antipsychotics	4.16 (1.52–11.38)	0.005*
Gliptins	3.32 (1.73–6.36)	<0.001*
Metformin	0.48 (0.18–1.28)	0.143

*Note*: Effects are expressed in the form of ORs with 95% CIs.

Abbreviation: BP, bullous pemphigoid; CI, confidence interval; OR, odds ratio.

* P < 0.05.

The results of BP association with any of the chronic diseases are detailed in Supporting Information Table S2. In BP patients there were fewer cases with CAD (14.1%) than in controls (30.0%), resulting in OR = 0.38 (95% CI 0.18–0.83, *P* = 0.012). Similarly, lower OR for functional thyroid disorder was found in BP patients (OR = 0.37, 95% CI 0.19–0.73, *P* = 0.003).

A multivariate logistic regression model was applied to establish relationships between BP as a dependent variable and chronic diseases that showed a relation with BP during univariate analyses (CAD, DM, and functional thyroid disorder). Table 3 shows an OR less than 1.0 for CAD (OR = 0.29, 95% CI 0.13–0.64, *P* = 0.002) and functional thyroid disorder (OR = 0.33, 95% CI 0.17–0.67, *P* = 0.002). On the contrary, DM had an effect on BP with an OR higher than 1.0 (OR = 1.98, 95% CI 1.04–3.76, *P* = 0.037).

**TABLE 3:** Multiple logistic regression model of BP with patients' chronic diseases that showed an association with BP as derived from univariate analyses.

	Method = enter	
	OR (95% CI)	*p*
CAD	0.29 (0.13–0.64)	0.002*
DM	1.98 (1.04–3.76)	0.037*
Functional thyroid disorder	0.33 (0.17–0.67)	0.002*

*Note*: Effects are expressed in the form of ORs with 95% CIs.

Abbreviations: BP, bullous pemphigoid; CAD, coronary artery disease; CI, confidence interval; DM, diabetes mellitus; OR, odds ratio.

* P < 0.05.

Consequently, two different models were applied. The first model includes the use of gliptins for diabetic patients (it also includes the use of statins and antipsychotics and no history of CAD or functional thyroid disorder) while the second model includes patients with DM with no use of gliptins (it also includes the use of statins and antipsychotics, and no history of CAD or functional thyroid disorder). Model 1 showed that BP was associated with gliptin use (OR = 3.52, 95% CI 1.79–6.93, *P* ≤ 0.001), statin use (OR = 3.90, 95% CI 1.77–8.63, *P* = 0.001), antipsychotic use (OR = 3.38, 95% CI 1.19–9.62, *P* = 0.022), no presence of CAD (OR = 4.15, 95% CI 1.19–9.62, *P* = 0.022), and no presence of functional thyroid disorder (OR = 2.83, 95% CI 2.33–6.04, *P* = 0.007). Model 2 showed similar results to model 1, but the presence of DM did not show any significant effect on BP (OR = 1.60, 95% CI 0.79–3.23, *P* = 0.188) (Table 4). As is evident from Table 4, statins and antipsychotics were independent risk factors for BP in diabetic patients and DM is not a risk factor in the absence of gliptins in diabetic patients.

**TABLE 4:** Multivariate logistic regression model of BP with drug categories and patients' medical history. Estimation on BP was expressed in the form of ORs (odds ratio) with 95%CIs (confidence intervals).

		OR (95% CI)	*P*
Model 1	Gliptins = yes	3.51 (1.79–6.93)	<0.001*
	Statins = yes	3.91 (1.77–8.63)	0.001*
	Antipsychotics = yes	3.38 (1.19–9.62)	0.022*
	CAD = no	4.15 (1.72–9.98)	0.001*
	Functional thyroid disorder = no	2.84 (1.33–6.04)	0.007*
Model 2	Statins = yes	4.73 (2.18–10.30)	<0.001*
	Antipsychotics = yes	3.31 (1.20–9.11)	0.021*
	CAD = no	3.94 (1.70–9.16)	0.003*
	Functional thyroid disorder = no	3.06 (1.46–6.40)	0.003*
	DM = yes	1.60 (0.79–3.23)	0.188

*Note*: Model 1: Explanatory variables: use of gliptins, statins, antipsychotics, non‐existence of CAD, functional thyroid disorder. Model 2: Explanatory variables: use of statins, antipsychotics, non‐existence of CAD, functional thyroid disorder, existence of DM. Estimation of BP was expressed in the form of ORs with 95% CIs.

Abbreviations: BP, bullous pemphigoid; CI, confidence interval; CAD, coronary artery disease; DM, diabetes mellitus; OR, odds ratio.

* P < 0.05.

**FIGURE 3:**
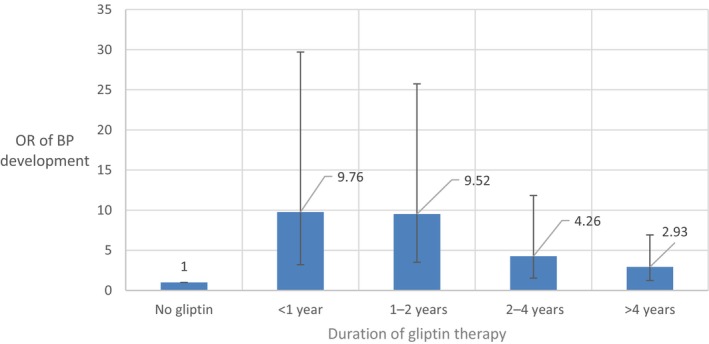
Estimated odds ratio (OR) with 95% confidence intervals of bullous pemphigoid (BP) development based on the time of gliptin intake: baseline (no gliptin intake), <1 year, 1–2 years, 2–4 years and >4 years. Blue bars indicate the OR and short horizontal bars indicate the upper 95% limit of the OR.

A person‐years approach was used to estimate the risk for BP after different durations of gliptin intake among various patients. In Figure 3, patients with BP are divided into groups of gliptin use duration (<1, 1–2, 2–4, and >4 years). The maximum duration of gliptin intake was 8 years. Similar odds ratios (ORs with 95% CI) were found during the first year of gliptin intake (OR = 9.76, 95% CI 6.55–19.95) and between the first and second year (OR = 9.52, 95% CI 6.00–16.22). Use of gliptins for a period of 2–4 years still shows an increased OR, but lower compared to those of the previous shorter time periods (OR = 4.26, 95% CI 2.73–7.57). The same was true for longer than 4 years of gliptin intake (OR = 2.93, 95% CI 1.69–3.99). Using Kaplan–Meier analysis, the median time for developing BP was 52.0 (95% CI 34.2–69.8) months or 4.3 (95% CI 2.9–5.8) years. (Figure S1). Multivariate logistic regression model of BP with gliptin intake duration, drug categories, and patients' medical history revealed similar OR magnitudes for BP development for any duration of gliptin intake except for gliptin intake period longer than 4 years (OR = 1.76, *P* = 0.260) (Table S4).

## 4 DISCUSSION

This case–control study indicates that drug‐induced BP can be associated with the use of specific drug categories, such as gliptins, statins, and antipsychotics. Chronic diseases, such as CAD and functional thyroid disorder, were less common in BP.

The study revealed an increased risk for BP development in patients taking gliptins and more specifically in those taking vildagliptin, linagliptin, and alogliptin. This finding confirms the study results demonstrated by Harano et al.,^10^ Hung et al.,^11^ and a meta‐analysis by Silverii et al.,^12^ in which DPP‐4i was associated with an increased risk of BP. Also, a systematic review and meta‐analysis performed by Phan et al.^13^ found a significant association between DPP‐4i use and BP. This association was stronger between vildagliptin and BP compared to linagliptin, and no association was found between sitagliptin and BP. Moreover, our study indicated that the risk for BP is higher during the first and second year of gliptin intake and remained higher across all time periods, which agrees with the finding of Kridin et al.^3^

The association of statins with the risk for BP development contrasts with the protective role demonstrated by Papadopoulou et al.^5^ and Rozenblat et al.^6^ However, Chang et al.^7^ showed that the risk of BP in statin users was not significantly different from that in nonusers. Murad et al.^14^ presented a case of rosuvastatin‐induced pemphigoid in a 58‐year‐old woman who was on no other medication.

The study analyses showed a relation between antipsychotics use and the development of BP. Neuroleptics, a specific category of antipsychotics, were firstly identified as independent risk factor for the development of BP by Bastuji‐Garin et al.^15^ Also, Varpuluoma et al.^8^ found at the drug‐class level that all butyrophenone derivates were significantly associated with an increased risk for BP. Moreover, a slightly increased risk of BP after exposure to antipsychotics was demonstrated in a meta‐analysis (meta‐OR = 1.79, 95% CI 1.17–2.73, I^2^ =0%).^16^

To the best of our knowledge, the exact pathophysiological mechanisms underlying these associations have not been completely elucidated. According to Chouchane et al.,^17^ in DPP‐4i‐induced BP most of theimmunoglobulin G (IgG) autoantibodies target preferentially epitopes in the mid portion of the extracellular domain of BP180, including the 120 kDA linear IgA dermatosis antigen (LAD‐1) and the C‐terminal domain, and not the juxtamembranous non‐ collagenous extracellular domain (NC16A) as in conventional BP. However, IgG autoantibodies against BP 180 NC16A have also been identified in certain cases of DPP‐4i‐induced BP. It has been hypothesized that these autoantibodies are the result of an epitope spreading phenomenon. Moreover, DPP‐4 is expressed in various immune cells such as cluster of differentiation 4 [CD4 (+)] and CD8 (+) T cells, B cells, macrophages, dendritic cells, and natural killer (NK) cells, and is able to modulate their functions. It is also a potent co‐stimulatory molecule in T‐cell signal transduction. Through its catalytic action, DPP‐4 can regulate the activity of several cytokines, peptide hormones, and chemokines, affecting molecules or signaling pathways pertaining to the immune system. Thus, the inactivation of DPP‐4 by DPP‐4i may potentially lead, among other effects, to the breakage of the immune tolerance for the basement membrane antigens, including BP180 and BP230.

Regarding the effect of statins on BP development, Murad et al.,^14^ who presented a case of rosuvastatin‐induced‐BP, also suggested the hypothesis that the offending drug may bind and modify protein molecules in the lamina lucida, uncovering some hidden antigenic site and potentially changing its antigenic properties. It may also cause dysregulation of T‐suppressor cell function which then releases antibodies, including those against the antibasement membrane zone that causes BP. Finally, the BP triggering mechanism of antipsychotics is also not fully understood. What is already known is that skin and nervous tissue derived from the same ectoderm and subtypes of the autoantigen BP230 have been identified in both the central and peripheral nervous system.^18^ It is supposed that antipsychotics may cause a cross‐reactive immune response towards neural and cutaneous antigens.

It has long been a scientific question of interest whether metformin is responsible for the induction of BP.^19^ Our results, similar to those of Molina Guarneros et al.,^20^ showed that metformin probably has no impact on BP. Moreover, our results are in accordance with the analysis performed by Kridin et al.^21^ in which the association of DPP‐4i use with BP was independent of the use of metformin.

Our study also examined the scientific question of whether gliptins or DM itself is responsible for the development of BP. The analysis in Table 3 shows that DM has a statistically significant association with the development of BP. However, according to the results presented in the subanalysis in Table 4, in the second model consisting of diabetic patients receiving no gliptins, DM had no impact on BP. This finding sheds more light on the association between BP and DM described previously by several other studies.^22^, ^23^

Our study showed no statistically significant difference between the cases and the controls regarding the prevalence of neurodegenerative diseases. Previous studies indicated an association between BP and neurodegenerative diseases/neurologic disorders,^24^, ^25^ but these studies analyzed a broader spectrum of neurologic disorders, including several other heterogenous neurological conditions such as cerebrovascular disease, seizures, stroke, hear loss etc. It is quite possible that the patients with these diagnoses had been taking drugs that might also be associated with BP development. Moreover, these studies investigated BP associations before the widespread use of gliptins, which may be also a diversifying factor. Due to the absence of similar findings from the literature, we consider the lower incidence of CAD and functional thyroid disorder observed in our study at first as a coincidental finding, until further verification by others.

One of the strengths of our study is the appropriate number of controls compared to cases. In addition, the study recruited cases and controls with Cretan ascendance, thus maintaining the homogeneity of the sample. A limitation of our study is its retrospective approach with a rather small number of cases. Furthermore, controls were randomly selected among hospital‐based individuals either admitted to the internal medicine department or visiting the dermatological and endocrinological outpatient clinics. Thus, they may eventually have more comorbidities and home medication in comparison to population‐based studies. Thus, we cannot exclude confounding bias.

In conclusion, our findings imply that specific drug classes, such as gliptins, statins, and antipsychotics, may be risk factors for the development of BP. In terms of gliptins, our study confirms the results of several other studies, albeit in a different sample, the Cretan population. Regarding the statins, this can be considered as a new finding because it contrasts with the few other published studies in the field. As regards the antipsychotics, our finding confirms the outcome of an up to now small number of studies. As the association of specific drug classes with the development of BP could radically change our current therapeutic approach in the daily medical practice, further research is needed to strengthen the evidence provided by the present study.

## FUNDING INFORMATION

K. Krasagakis has received grants from Eli Lilly, Faran, and LEO Pharma; payment or honoraria for lectures, presentations, or educational events from Eli Lilly, Galderma, and UCB; support for attending meetings and/or travel from AbbVie, Boderm, Eli Lilly, Frezyderm, Galderma, Galenica, Genesis, Janssen, LEO Pharma, Pfizer, Sanofi, and UCB; has participated on the Advisory Bοard for Eli Lilly, Boehringer Ingelheim, Sanofi, and UCB; and is or has been an investigator in clinical trials for AiCuris, Amgen, Eli Lilly, Janssen, LEO Pharma, and Novartis. S. Kruger‐Krasagakis has received honoraria for lectures, presentations, or educational events from Galderma and UCB; support for attending meetings and/or travel from AbbVie, Eli Lilly, Galderma, Genesis, LEO Pharma, Pfizer, and UCB; and has participated on the Advisory Board for Galderma, UCB, Janssen, and AbbVie. E. Kavvalou, G. Chlouverakis, P. Xekouki, V. Daraki, C. Kouvidou, and E. Lagoudaki have no financial or non‐financial conflicts of interest to declare.

## CONFLICT OF INTEREST STATEMENT

All authors meet the three criteria for authorship as recommended. The authors did not receive payment related to the development of this manuscript.

## ETHICS STATEMENT

Approval of the research protocol by an Institutional Reviewer Board: The study was conducted in accordance with the Declaration of Helsinki and the protocol was approved by the University of Crete and the University General Hospital of Heraklion (Protocol Numbers 156/30.07.2020 and 1005/33/02‐12‐2020, respectively).

## Supporting information


Data S1.
