# Supplementary material for: Association of statins, gliptins, and antipsychotics with bullous pemphigoid: A case–control study in the Cretan population
Source: J Dermatol. 2025 Jan 8;52(2):291–8. doi: 10.1111/1346-8138.17603 (PMC11807367; doi:10.1111/1346-8138.17603)
Supplement: Supplementary file 1 — Data S1. [file JDE-52-291-s001.docx]

**SUPPLEMENTARY MATERIAL**

**Table 1S.** Association of BP with various drug categories expressed in the form of ORs (odds ratio) with 95%CIs (confidence intervals). Each line indicates the association of BP (yes/no) as dependent with a drug category; n(%) refers to frequency and %frequency respectively. Estimated ORs with 95%CIs and p-values as resulted from logistic regression analysis.

|  | **Group** | |  | | |
| --- | --- | --- | --- | --- | --- |
|  | **Control** | **BP** | **Total** | **OR (95%CI)** | **p** |
|  | **n (%)**191 | **n (%)**64 | **n** |  |  |
| **PPIs^†^** | 86 (46.7) | 32 (50.8) | 229 | 1.18 (0.66-2.09) | 0.578 |
| **β – blockers** | 56 (30.4) | 22 (34.9) | 244 | 1.23 (0.67-2.25) | 0.509 |
| **Statins** | 99 (53.8) | 52 (82.5) | 244 | 4.06 (1.99-8.27) | <0.001* |
| **ARBs**^‡^ | 97 (52.7) | 33 (52.4) | 244 | 0.99 (0.56-1.75) | 0.963 |
| **ChEIs**^§^ | 10 (5.4) | 6 (9.5) | 244 | 1.83 (0.64-5.26) | 0.255 |
| **Bisphosphonates** | 26 (14.1) | 8 (12.7) | 244 | 0.88 (0.38-2.07) | 0.776 |
| **ACE**^¶^ **inhibitors** | 49 (26.6) | 15 (23.8) | 244 | 0.86 (0.44-1.68) | 0.659 |
| **5-ARIs**^±^ | 13 (7.1) | 5 (7.9) | 244 | 1.13 (0.39-3.32) | 0.818 |
| **α-Blockers** | 31 (16.8) | 7 (11.1) | 244 | 0.62 (0.26-1.48) | 0.276 |
| **CCBs**^ꬹ^ | 83 (45.1) | 28 (44.4) | 244 | 0.97 (0.55-1.73) | 0.927 |
| **Antipsychotics** | 11 (6.0) | 11 (17.5) | 244 | 3.33 (1.36-8.11) | 0.006* |
| **Antidepressants** | 16 (8.7) | 4 (6.3) | 244 | 0.71 (0.23-2.22) | 0.556 |
| **Dopaminergic Agents** | 3 (1.6) | 4 (6.3) | 244 | 4.09 (0.89-18.81) | 0.051 |
| **Diuretics** | 93 (50.5) | 31 (49.2) | 244 | 0.95 (0.54-1.68) | 0.855 |
| **H2**^∞^ **Antagonists** | 22 (12.0) | 7 (11.1) | 244 | 0.92 (0.37-2.27) | 0.857 |
| **Antithrombotic agents** | 95 (51.6) | 37 (57.8) | 245 | 1.28 (0.72-2.28) | 0.393 |
| **Antidiabetic drugs** | 90 (47.1%) | 45 (70.3%) | 248 | 2.47 (1.34-4.55) | 0.003* |

* p < 0.05
**†** Proton pump inhibitors
‡ Angiotensin receptor blockers
§ Cholinesterase inhibitors
¶ Angiotensin-converting enzyme
± 5a- Reductase inhibitors
ꬹ Calcium channel blockers
∞ Histamine type 2 receptor antagonists

**Table 2S.** Association of BP with medical history diseases of BP patients and controls. Association were expressed in the form of OR (odds ratio) with 95%CIs (confidence intervals) as resulted from simple logistic regression; n(%) refers to frequency and %frequency respectively.

|  | **Control** | **BP** | **OR (95%CI)** | **p** |
| --- | --- | --- | --- | --- |
|  | **n (%)** | **n (%)** |  |  |
| **Arterial Hypertension** | 144 (75.8) | 45 (70.3) | 0.82 (0.43-1.54) | 0.533 |
| **CAD^†^** | 57 (30.0) | 9 (14.1) | 0.38 (0.18 - 0.83) | 0.012* |
| **DM**^‡^ | 105 (56.1) | 46 (71.9) | 2.00 (1.08 - 3.70) | 0.027* |
| **Dyslipidemia** | 120 (63.2) | 48 (75.0) | 1.75 (0.92 - 3.31) | 0.083 |
| **COPD**^§^ | 29 (15.2) | 4 (6.3) | 0.37 (0.13 - 1.10) | 0.065 |
| **Ca**^¶^ | 39 (20.4) | 8 (12.5) | 0.56 (0.25 - 1.26) | 0.157 |
| **Atopia** | 54 (28.9) | 13 (20.3) | 0.63 (0.32 - 1.25) | 0.181 |
| **Functional thyroid disorder** | 77 (40.5) | 13 (20.3) | 0.37 (0.19 - 0.73) | 0.003* |
| **Neurodegenerative diseases** | 40 (21.2) | 14 (21.9) | 1.04 (0.52 - 2.07) | 0.904 |
| **Psychiatric diseases** | 41 (21.7) | 14 (21.9) | 1.01 (0.51 - 2.01) | 0.976 |
| **Hyperuricemia** | 33 (17.4) | 12 (18.8) | 1.10 (0.53 - 2.28) | 0.802 |
| **Autoimmune diseases** | 54 (28.3) | 16 (25.0) | 0.85 (0.44 - 1.62) | 0.621 |
| **Psoriasis** | 10 (5.2) | 3 (4.7) | 0.89 (0.24 - 3.34) | 0.863 |
| **Ophthalmologic diseases** | 42 (22.2) | 11 (17.2) | 0.73 (0.35 - 1.51) | 0.392 |

*p < 0.05 **†** Coronary artery disease
‡ Diabetes mellitus
§ Chronic obstructive pulmonary disease
¶ Cancer

**Table 3S**. Association of BP with the use of various drugs expressed in the form of OR (odds ratio) with 95%CIs (confidence intervals); n(%) refers to frequency and %frequency respectively.

|  | **Group** | | | |
| --- | --- | --- | --- | --- |
|  | **Control** | **BP** | **OR (95%CI)** | **p** |
|  | **n (%)** | **n (%)** |  |  |
| **Statins** | 99 (53.8) | 52 (82.5) | 4.06 (1.99 - 8.27) | <0.001* |
| **Atorvastatin** | 63 (34.2) | 31 (49.2) | 1.86 (1.04 - 3.32) | 0.035* |
| **Simvastatin** | 38 (20.7) | 14 (22.2) | 1.1 (0.55 - 2.2) | 0.792 |
| **Pitavastin** | 2 (1.1) | 3 (4.8) | 4.55 (0.74 - 27.88) | 0.074 |
| **Rosuvastatin** | 12 (6.5) | 7 (11.1) | 1.79 (0.67 - 4.77) | 0.238 |
| **Pravastatin** | 5 (2.7) | 3 (4.8) | 1.79 (0.42 - 7.72) | 0.429 |
| **Antipsychotics** | 11 (6) | 11 (17.5) | 3.33 (1.36 - 8.11) | 0.006* |
| **Aripiprazole** | 0 (0) | 1 (1.6) | *0.25 (0.2 - 0.31) | 0.087 |
| **Olanzapine** | 4 (2.2) | 1 (1.6) | 0.73 (0.08 - 6.62) | 0.775 |
| **Perphenazine** | 2 (1.1) | 1 (1.6) | 1.47 (0.13 - 16.47) | 0.754 |
| **Quetiapine** | 7 (3.8) | 9 (14.3) | 4.21 (1.5 - 11.85) | 0.004* |
| **Haloperidol** | 0 (0) | 1 (1.6) | *0.25 (0.2 - 0.31) | 0.087 |
| **Gliptins** | 57 (31) | 43 (67.2) | 4.27 (2.33 - 7.83) | <0.001* |
| **Linagliptin** | 5 (2.7) | 10 (15.6) | 6.63 (2.17 - 20.23) | <0.001* |
| **Vildagliptin** | 36 (19.6) | 28 (43.8) | 3.2 (1.73 - 5.91) | <0.001* |
| **Alogliptin** | 3 (1.6) | 5 (7.8) | 5.11 (1.19 - 22.04) | 0.016* |
| **Sitagliptin** | 20 (10.9) | 9 (14.1) | 1.33 (0.57 - 3.1) | 0.503 |
| **Saxagliptin** | 6 (3.3) | 2 (3.1) | 0.96 (0.19 - 4.87) | 0.958 |
| **Insulin** | 26 (14.1) | 12 (18.8) | 1.4 (0.66 - 2.98) | 0.377 |
| **Insulin glargine** | 13 (7.1) | 6 (9.4) | 1.35 (0.49 - 3.72) | 0.557 |
| **Insulin aspart** | 11 (6) | 2 (3.1) | 0.51 (0.11 - 2.35) | 0.378 |
| **Human insulin** | 3 (1.6) | 3 (4.7) | 2.97 (0.58 - 15.09) | 0.17 |
| **Insulin degludec** | 7 (3.8) | 0 (0) | *0.73 (0.68 - 0.79) | 0.113 |
| **Insulin detemir** | 2 (1.1) | 1 (1.6) | 1.44 (0.13 - 16.2) | 0.764 |
| **Insulin lispro** | 1 (0.5) | 1 (1.6) | 2.9 (0.18 - 47.13) | 0.432 |
| **Metformin** | 76 (41.3) | 39 (60.9) | 2.22 (1.24-3.97) | 0.007* |
| **Gliclazide** | 25 (13.6%) | 12 (18.8) | 1.47 (0.69-3.13) | 0.318 |
| **Pioglitazone** | 5 (2.7%) | 5 (7.8) | 3.03 (0.85-10.85) | 0.074 |
| **Liraglutide** | 4 (2.2) | 0 (0.0) | *0.74 (0.69-0.80) | 0.234 |

*p < 0.05

**Table 4S.** Multivariate logistic regression model of BP with gliptin intake duration, drug categories and patients’ medical history. Estimation on BP was expressed in the form of ORs (odds ratio) with 95%CIs (confidence intervals).

|  | **OR (95%CI)** | **p** |
| --- | --- | --- |
| **No gliptin** | 1.00 | <0.001 |
| **Gliptin intake < 1 year** | 11.51 (3.20-41.39) | <0.001* |
| **Gliptin intake 1-2 years** | 10.96 (3.40- 35.35) | <0.001* |
| **Gliptin intake 2-4 years** | 5.25 (1.61- 17.17) | 0.006* |
| **Gliptin intake >4 years** | 1.76 (0.66- 4.72) | 0.260 |
| **Statins = yes** | 4.68 (1.98- 11.07) | <0.001* |
| **Antipsychotics =yes** | 5.13 (1.60- 16.48) | 0.006* |
| **CAD^†^ =no** | 6.22 (2.24- 17.25 | <0.001* |
| **Functional thyroid disorder= no** | 3.39 (1.45- 7.91) | 0.005* |

*p < 0.05
 **†** Coronary artery disease

**Figure 1S**

**
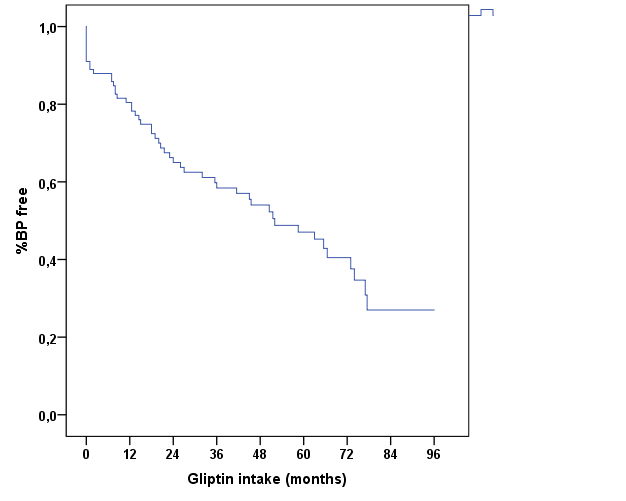
**
